# Economic burden of hospital-acquired multidrug-resistant organism infections in patients undergoing gastrointestinal surgery: a bootstrap-based retrospective cohort study

**DOI:** 10.3389/fpubh.2026.1886953

**Published:** 2026-09-10

**Authors:** Peng Yan, Chunlin Cai, Xiaofang Liu, Liping Jiang, Yangzhen Liu

**Affiliations:** 1 Department of Interventional Vascular Surgery, Hunan Provincial People’s Hospital and The First Affiliated Hospital of Hunan Normal University, Changsha, Hunan Province, China; 2 Department of Hospital Infection Management, The First Hospital of Changsha, Changsha, Hunan Province, China; 3 Hospital Administration Office, The First Hospital of Changsha, Changsha, Hunan Province, China

**Keywords:** economic burden, gastrointestinal surgery, hospital-acquired infection, ICU length of stay, multidrug-resistant organisms

## Abstract

**Background:**

Multidrug-resistant organism (MDRO) infections impose substantial clinical and economic burdens on surgical patients. However, specialty-specific analyses remain limited. This study quantified the economic impact of MDRO hospital-acquired infections (HAIs) on patients undergoing gastrointestinal surgery.

**Methods:**

100 patients with postoperative HAIs at a tertiary hospital (January 2022–December 2024) were stratified into MDRO (*n* = 41) and no-MDRO (*n* = 59) groups. Demographic, surgical, infection data and economic indices were collected via hospital’s electronic medical record system, laboratory information system and BlueDragonfly HAI surveillance platform. Statistical analyses were performed using R 4.4.2 software. Based on the analysis of baseline data, significant differences were observed in ICU-related costs, such as Total hospitalization costs, Laboratory diagnostics, Imaging diagnostics, Clinical diagnostic procedures, Nursing care, Disposable therapeutic materials, and Nonsurgical treatments. We hypothesized that prolonged ICU length of stay is associated with increased costs. Considering that the original sample size is relatively small, we adopt the bootstrap resampling method to increase the sample size. A non-parametric bootstrap approach was used to perform probabilistic sensitivity analysis (PSA) to quantify the uncertainty in incremental costs (ΔC) and incremental effects (ΔE). The cost-effectiveness acceptability curve (CEAC) was based on the net monetary benefit (NMB = WTP × ΔE−ΔC), where WTP denotes the willingness-to-pay threshold (in CNY per additional ICU length of stay).

**Results:**

The MDRO had significantly longer ICU stay (median 2 vs. 0 days; *p* < 0.001), durations of antibiotic therapy (median 25 vs. 23 days; *p* = 0.017) and hospitalization durations (median 35 vs. 29 days; *p* = 0.030). Treatment outcomes differed significantly between groups (*p* < 0.05). Total hospitalization costs were substantially higher in the MDRO (median 85,912.86 vs. 64,746.75 CNY; *p* < 0.05). Incremental cost (ΔC, 95% CI: 18,405–78,031 CNY) and incremental effect (ΔE, 95% CI: 3.09–8.51 days in ICU length of stay) comparing MDRO with no-MDRO. Albeit with notable uncertainty, the 95% confidence ellipse lies essentially in the first quadrant, suggesting that the direction of the cost-effectiveness difference is consistent (higher cost and longer ICU length of stay). WTP thresholds: median ICER (8,376 CNY, 95% CI:5,081–10,864 CNY, probability:39.9%). Significant cost increases, in descending order, were observed for laboratory diagnostics, nonsurgical treatments, clinical diagnostic procedures, nursing care, imaging diagnostics and disposable therapeutic materials (all *p* < 0.05).

**Conclusion:**

The study demonstrate that HAIs caused by MDROs in gastrointestinal surgery patients exacerbate medical resource consumption and significantly increase hospitalization costs, prolonging hospital stay duration, ICU length of stay, and duration of antibiotic therapy. Consequently, these infections impose a significant economic burden, with laboratory diagnostics, non-surgical treatments and clinical diagnostic procedures identified as the major cost drivers. We fully acknowledge that, as a retrospective observational study, comparing the MDRO and non-MDRO groups cannot establish that MDRO infection caused the increased costs, without adequate adjustment for confounding factors (e.g., severity of underlying illness, age, length of hospital stay prior to ICU admission).

## Introduction

Multidrug-resistant organism (MDRO) infections represent a critical global public health challenge, with antimicrobial resistance (AMR) projected to cause 8.22 million deaths annually by 2050 (1). Surgical patients undergoing gastrointestinal procedures face increased MDRO infection risks due to extensive tissue trauma, postoperative immunosuppression, and gut microbiota translocation (2). These infections substantially increase mortality (e.g., 40 ~ 50% fatality rates in carbapenem-resistant *Klebsiella pneumoniae* infections (3)) and impose severe economic burdens through prolonged hospitalization, expensive antibiotic regimens, and additional resource utilization (4).

Existing evidence confirms that hospital-acquired infections (HAIs) caused by AMR significantly increase the costs and the length of hospital stay (5). In gastrointestinal surgery, surgical site infection rates range from 5.3 to 19.5%, with MDROs accounting for a considerable proportion (6, 7). Reoperations, intensive care needs, and extended antibiotic therapy for MDRO infections increase the average hospitalization costs by 26% (8). Notably, multidrug-resistant Enterobacteriaceae (e.g., ESBL-producing *Escherichia coli* and carbapenem-resistant Enterobacteriaceae) are frequently detected in patients undergoing gastrointestinal surgery (7). These pathogens employ resistance mechanisms such as *β*-lactamase production (9), necessitating costly third-line antibiotics (e.g., tigecycline and polymyxin) (10) at 1.8-fold the daily cost of first-line agents (11).

Prior studies have consistently demonstrated that MDRO infections are associated with higher healthcare costs compared to non-MDRO infections, particularly in intensive care settings. However, most of these studies have focused on general ICU populations or specific infection types (e.g., bloodstream infections, ventilator-associated pneumonia). Few studies have specifically examined the economic burden of MDRO infections in gastrointestinal surgery patients – a population with unique characteristics, including gastrointestinal tract disruption, prolonged perioperative antibiotic exposure, and high rates of intra-abdominal infections, all of which may predispose them to MDRO colonization and infection. Gastrointestinal surgery patients are particularly vulnerable to MDRO infections due to the frequent need for broad-spectrum antibiotic prophylaxis, prolonged operative times and postoperative intensive care. Moreover, postoperative intra-abdominal infections caused by MDROs often require prolonged ICU stays, repeated surgical interventions and escalated antibiotic therapy—all of which may substantially drive up costs. While, data quantifying this incremental economic impact remain scarce. To our knowledge, this is an uncommon study to systematically compare ICU-related costs between MDRO and non-MDRO groups specifically in a gastrointestinal surgery cohort, with a detailed analysis of cost drivers. By focusing on this understudied population, our findings provide targeted economic evidence that can inform infection prevention strategies and resource allocation in gastrointestinal surgical wards. More effective therapies may yield considerable cost savings, which would support the rollout of novel therapeutic strategies [e.g., phage therapy (12) and photodynamic treatment (13)].

## Methodology

### Study population

This retrospective cohort study included 100 patients with postoperative HAIs who underwent gastrointestinal surgery at a tertiary Grade 3A hospital in Changsha, China, between January 1, 2022, and December 31, 2024. The patients were identified from the hospital’s electronic medical record system, laboratory information system, and BlueDragonfly HAI surveillance platform. All eligible patients during 2022–2024 were included consecutively. The participants were stratified into two cohorts based on causative pathogens: (1) the MDRO group (*n* = 41), comprising patients with infections caused by multidrug-resistant organisms, and (2) the no-MDRO group (*n* = 59), comprising patients with infections caused by non-MDR pathogens.

### Inclusion and exclusion criteria

Eligible participants met all the following criteria: (1) diagnosed with HAI according to the *Diagnostic Criteria for Nosocomial Infections (Trial)* (14); (2) aged ≥18 years and had undergone elective or emergency gastrointestinal surgery; (3) laboratory-confirmed postoperative HAI; and (4) complete medical expense records, microbiological reports, and clinical documentation available. Patients were excluded if they (1) developed community-acquired infections or had no evidence of postoperative HAI; (2) had primary immunodeficiency disorders or received immunosuppressive therapy within 30 days preoperatively; or (3) presented critical missing data preventing comprehensive analysis. All inclusion/exclusion criteria were independently verified by two infection preventionists, and discrepancies were resolved through the third one.

### Data collection and processing

Multisource data integration was performed using the hospital’s electronic medical record system, laboratory information system, and BlueDragonfly HAI surveillance platform. The collected variables included (1) demographic characteristics (age, sex and comorbidities); (2) surgical parameters (open/laparoscopic approach, elective/emergency status, operative duration, and wound healing grade); (3) infection metrics (site of infection, pathogen type, antimicrobial resistance profile, and duration of antibiotic therapy); and (4) economic burden indicators (hospital stay, Total hospitalization costs (indirect costs excluded), and ICU length of stay). Demographic, surgical, and infection data were subjected to independent dual-entry verification using EpiData 3.1, with cross-verification for discrepancies. Economic indices were extracted as structured datasets from the hospital’s billing system. All data dimensions were linked using unique patient hospitalization identifiers for deterministic matching. No critical data were missing.

### Pathogen identification and MDRO classification

Microbiological samples from postoperative infection sites (e.g., wound exudates, peritoneal drainage fluid and blood) were aseptically collected per clinical protocols and processed within 2 h of collection. For isolation, standard culture techniques followed by matrix-assisted laser desorption/ionization time-of-flight mass spectrometry (MALDI-TOF MS; bioMérieux) were used for rapid species confirmation. Antimicrobial susceptibility testing against *β*-lactams, carbapenems, fluoroquinolones, and aminoglycosides was performed using the VITEK 2 Compact automated system (bioMérieux) or Kirby-Bauer disk diffusion, which strictly adhered to Clinical and Laboratory Standards Institute performance standards (CLSI M100-S31, 2021) (15). Multidrug resistance was defined as nonsusceptibility to 3 or more antimicrobial categories proposed by Magiorakos et al. (16). Patients with polymicrobial infections were included in the analysis. For classification, a patient was assigned to the MDRO group if at least one of the isolated pathogens met the predefined MDRO criteria (nonsusceptibility to ≥3 antimicrobial categories). If none of the isolated organisms met the MDRO criteria, the patient was assigned to the non-MDRO group.

### Statistical analysis

Statistical analyses were performed using the R software (version 4.4.2) and figures were generated with the ggplot2 package. Categorical data were presented as frequencies and proportions, and comparisons between groups for categorical variables were conducted using the chi-square test. Nonnormally distributed data are expressed as medians (interquartile ranges), and intergroup comparisons were analysed using the Wilcoxon rank-sum test. Considering that the original sample size is relatively small, we adopt the bootstrap resampling method to increase the sample size. A non-parametric bootstrap approach was used to perform probabilistic sensitivity analysis (PSA) to quantify the uncertainty in incremental costs (ΔC) and incremental effects (ΔE). For both groups (MDRO and no-MDRO), 5,000 resamples were drawn with replacement, each of the same size as the original sample. For each bootstrap replication, we calculated the ΔC as the difference in median total hospitalization costs between MDRO and no-MDRO, and the ΔE as the difference in median ICU length of stay. The bootstrap replicates were used to generate a cost-effectiveness scatter plot. A non-parametric 95% confidence ellipse based on Mahalanobis distance percentiles was constructed. The incremental cost-effectiveness ratio (ICER) was defined as ΔC/ΔE. Because the sampling distribution of the ICER is often skewed, the median was used as the point estimate. The cost-effectiveness acceptability curve (CEAC) was based on the net monetary benefit (NMB = WTP × ΔE−ΔC), where WTP denotes the willingness-to-pay threshold (in CNY per additional ICU length of stay). The 95% CI for this probability was obtained using the bootstrap percentile method. A two-tailed *p* value of less than 0.05 was considered statistically significant. The work has been reported in line with the STROCSS criteria (17).

## Results

### Patient baseline characteristics

The study cohort comprised 100 patients with postoperative HAIs following gastrointestinal surgery, with 41 patients (41%) in the MDRO group and 59 patients (59%) in the no-MDRO group. The baseline data revealed no statistically significant differences between the two groups in terms of sex distribution or median age (*p > 0.05*). In the analysis of comorbidities, no statistically significant differences were observed between the MDRO group and the no-MDRO group in terms of the incidence of diabetes, hypertension, or malignancy (*p* > 0.05) (Table 1).

**Table 1:** Baseline characteristics of patients.

Variable	Overall (*n* = 100^1^)	Non-MDRO group (*n* = 59^1^)	MDRO group (*n* = 41^1^)	Statistic^2^	*p* value^2^	SMD
Sex, *n* (%)				0.073	0.79	0.10
Male	68 (68%)	39 (66%)	29 (71%)			
Female	32 (32%)	20 (34%)	12 (29%)			
Age (years)	67.00 (57.50, 73.00)	66.00 (55.00, 71.00)	70.00 (60.00, 74.00)	1,017	0.18	−0.25
Diabetes, *n* (%)				2.08	0.15	0.34
Yes	21 (21%)	9 (15%)	12 (29%)			
No	79 (79%)	50 (85%)	29 (71%)			
Hypertension, *n* (%)				0.388	0.53	0.17
Yes	29 (29%)	19 (32%)	10 (24%)			
No	71 (71%)	40 (68%)	31 (76%)			
Malignancy, *n* (%)				0.002	0.97	0.05
Yes	60 (60%)	36 (61%)	24 (59%)			
No	40 (40%)	23 (39%)	17 (41%)			

^1^*n* (%); Median (IQR).

^2^Pearson’s Chi-squared test; Wilcoxon rank sum test.

### Comparison of surgical characteristics, resource utilization, and treatment outcomes among patients

Analysis of the surgical characteristics revealed no statistically significant differences between the MDRO group and no-MDRO group in terms of operative duration, distribution of wound healing grades, proportion of emergency surgeries, or proportion of laparoscopic surgeries (*p* > 0.05). However, significant differences in treatment outcomes and resource utilization were observed between the two groups. Compared with the no-MDRO group, patients in the MDRO group had a longer ICU length of stay (*p < 0.001*), more total days of antimicrobial use (*p = 0.017*), and a longer hospital stay (*p = 0.030*). In addition, the MDRO group had a higher mortality rate and lower cure rate, with a statistically significant difference in the treatment outcomes of the primary diagnosis between the two groups (*p = 0.013*) (Table 2).

**Table 2:** Comparison of surgical characteristics, resource consumption and treatment outcomes among patients.

Variable	Overall (*n* = 100^1^)	Non-MDRO group (*n* = 59^1^)	MDRO group (*n* = 41^1^)	Statistic^2^	*p* value^2^
Operative duration (min)	220.00 (155.00, 254.00)	220.00 (165.00, 254.00)	220.00 (146.00, 279.00)	1,066	0.82
Emergency surgery, *n* (%)				0.065	0.80
Yes	22 (22%)	14 (24%)	8 (20%)		
No	78 (78%)	45 (76%)	33 (80%)		
Laparoscopic surgery, *n* (%)				0.961	0.33
Yes	51 (51%)	33 (56%)	18 (44%)		
No	49 (49%)	26 (44%)	23 (56%)		
Wound healing grade, *n* (%)				5.42	0.61
I/A	3 (3.0%)	1 (1.7%)	2 (4.9%)		
II/A	17 (17%)	11 (19%)	6 (15%)		
III/A	59 (59%)	37 (63%)	22 (54%)		
II/B	2 (2.0%)	1 (1.7%)	1 (2.4%)		
II/C	2 (2.0%)	2 (3.4%)	0 (0%)		
III/B	6 (6.0%)	2 (3.4%)	4 (9.8%)		
III/C	6 (6.0%)	3 (5.1%)	3 (7.3%)		
III/Other	5 (5.0%)	2 (3.4%)	3 (7.3%)		
ICU length of stay (days)	0.00 (0.00, 3.50)	0.00 (0.00, 1.00)	2.00 (0.00, 10.00)	746	<0.001
Antibiotic therapy duration (days)	24.00 (17.50, 33.00)	23.00 (16.00, 28.00)	25.00 (19.00, 37.00)	869	0.017
Hospital stay (days)	31 (25, 41)	29 (25, 37)	35 (27, 49)	899	0.030
Treatment outcome of primary diagnosis at discharge, *n* (%)				8.65	0.013
Cured	78 (78%)	51 (86%)	27 (66%)		
Improved	18 (18%)	8 (14%)	10 (24%)		
Died	4 (4.0%)	0 (0%)	4 (9.8%)		

^1^*n* (%); Median (IQR).

^2^Pearson’s Chi-squared test; Wilcoxon rank sum test.

### Cost-effectiveness analysis

The bootstrap analysis showed that, between the no-MDRO and the MDRO, there were a median increase in total hospitalization costs of 46,973 CNY (95% CI: 18,405–78,031CNY) and a median increase in ICU length of stay of 5.59 days (95% CI: 3.09–8.51 days).

**Figure 1:**
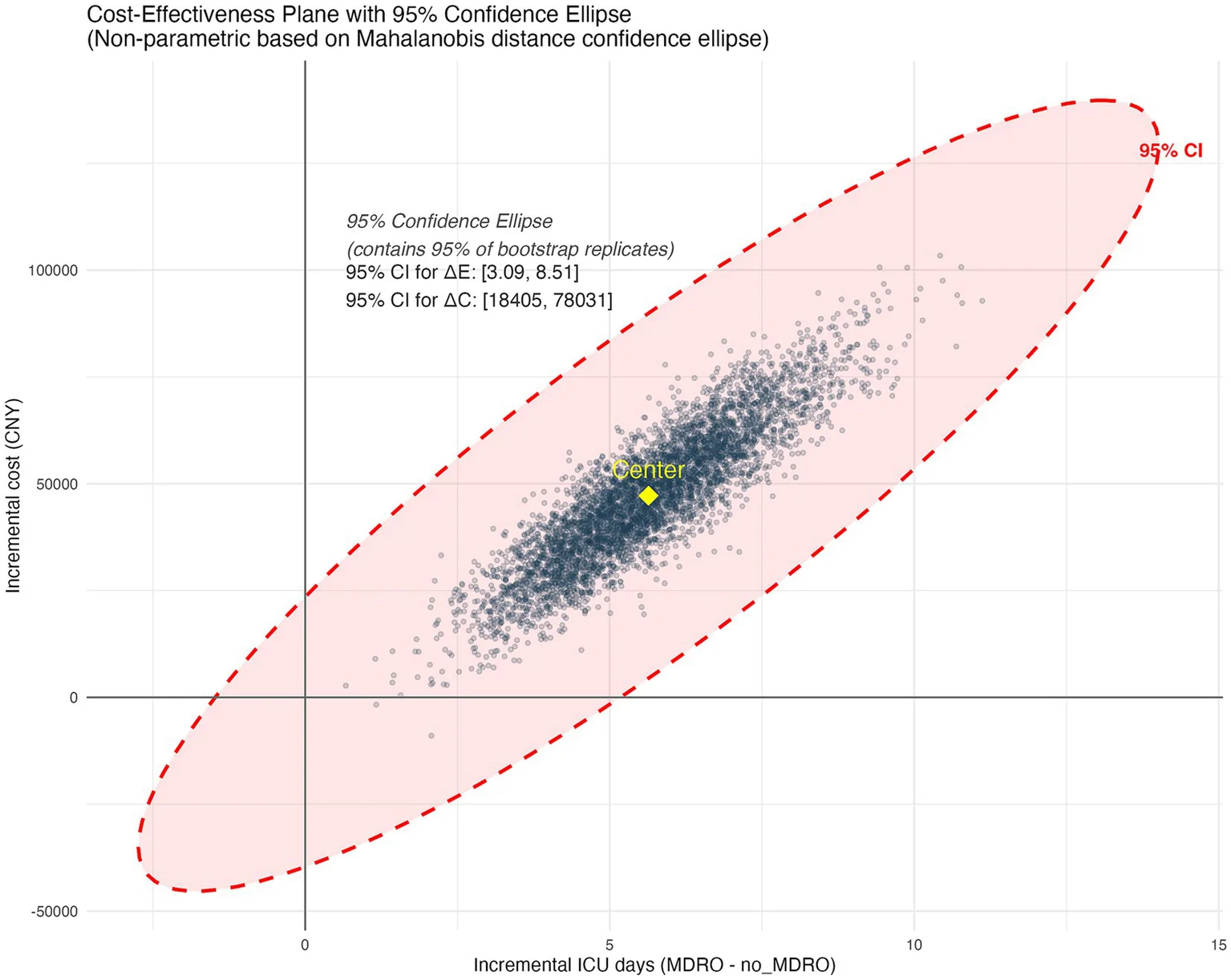
Cost-effectiveness plane of MDRO versus no-MDRO. Each grey point represents one bootstrap replicate (*n* = 5,000) of the incremental cost (*ΔC*, 95% CI: 18405–78,031 CNY) and incremental effect (*ΔE*, 95% CI: 3.09–8.51 days in ICU length of stay) comparing MDRO with no-MDRO. The yellow diamond (Center) indicates the median incremental cost and median incremental effect. The red dashed ellipse is the non-parametric 95% confidence ellipse constructed using the 95th percentile of the Mahalanobis distances of the bootstrap replicates from their centroid. The horizontal and vertical dashed lines represent *ΔC* = 0 and *ΔE* = 0, respectively. Points in the north-east quadrant indicate that MDRO is more costly and associated with longer ICU stay. Albeit with notable uncertainty, the 95% confidence ellipse lies essentially in the first quadrant, suggesting that the direction of the cost-effectiveness difference is consistent (higher cost and longer ICU length of stay).

The cost-effectiveness plane (Figure 1) shows that the vast majority of bootstrap scatter points lie in the north-east quadrant (ΔC > 0, ΔE > 0), indicating that the MDRO is both more costly and associated with longer ICU length of stays compared to the no-MDRO. Albeit with notable uncertainty, the 95% confidence ellipse lies essentially in the first quadrant, suggesting that the direction of the cost-effectiveness difference is consistent (higher cost and longer ICU length of stay).

**Figure 2:**
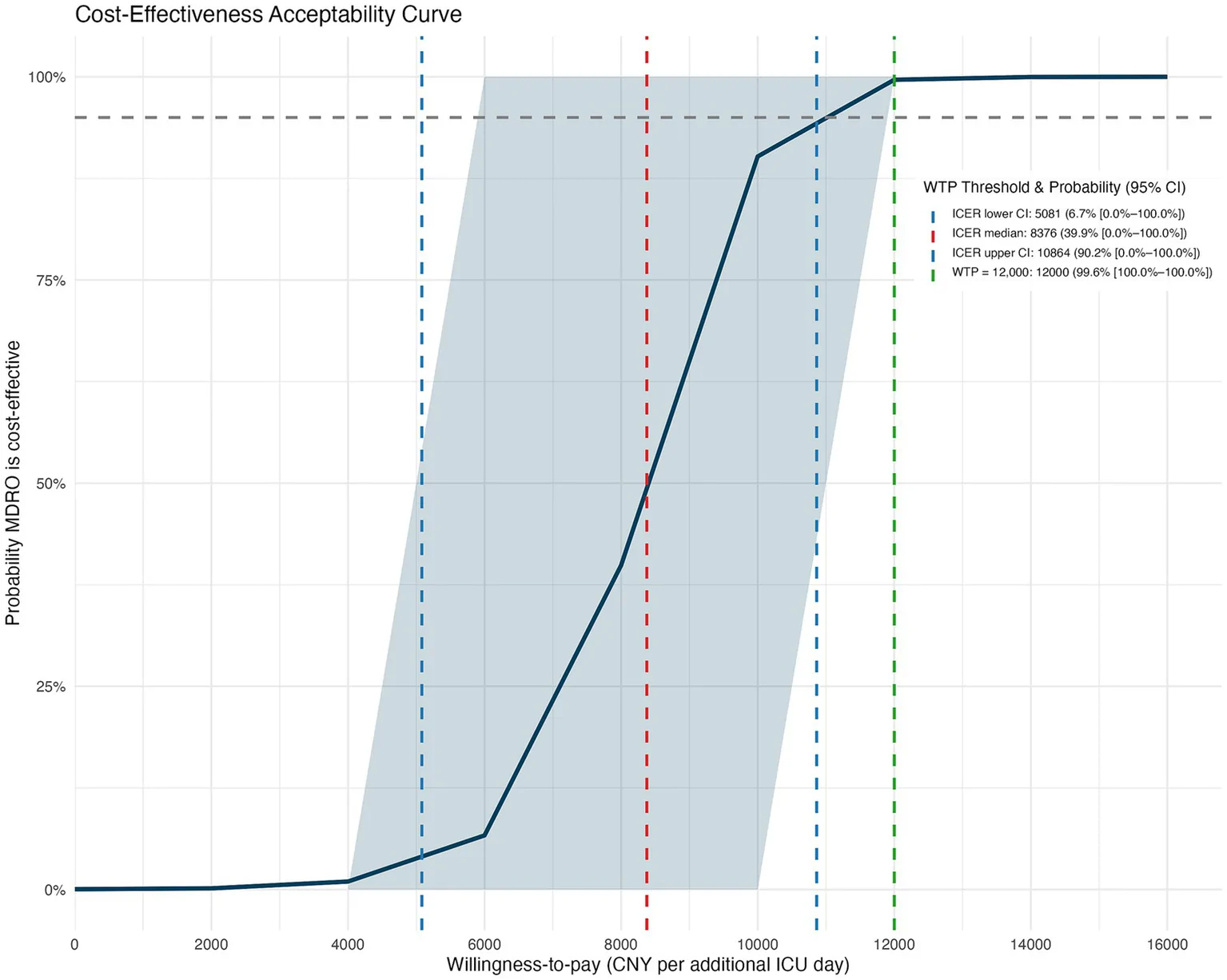
Cost-effectiveness acceptability curve for MDRO versus no-MDRO. The solid blue line shows the probability that the MDRO group is cost-effective as a function of the willingness-to-pay (WTP) threshold per additional ICU stay (CNY). The light blue ribbon represents the 95% CI obtained by the bootstrap percentile method. The grey dashed horizontal line indicates the 95% probability reference level. Vertical dashed lines (with corresponding labels) are shown at selected WTP thresholds: ICER lower 95% CI (5,081 CNY, probability: 6.7%), median ICER (8,376 CNY, probability: 39.9%), ICER upper 95% CI (10,864 CNY, probability: 90.2%), and 12,000 CNY. At a WTP threshold of 12,000 CNY per additional ICU length of stay, the probability rises to about 99.6%.

The cost-effectiveness acceptability curve (Figure 2) shows that when a willingness-to-pay threshold equal to the median ICER (8,376 CNY per additional ICU length of stay; 95% CI: 5,081–10,864 CNY per additional ICU length of stay), the probability of being attributed to MDRO is approximately 39.9% (probability of lower to upper: 6.7–90.2%). When the WTP threshold increases to 12,000 CNY per additional ICU length of stay, this probability rises to about 99.6%.

### Intergroup differences in various hospitalization expenses of patients

**Figure 3:**
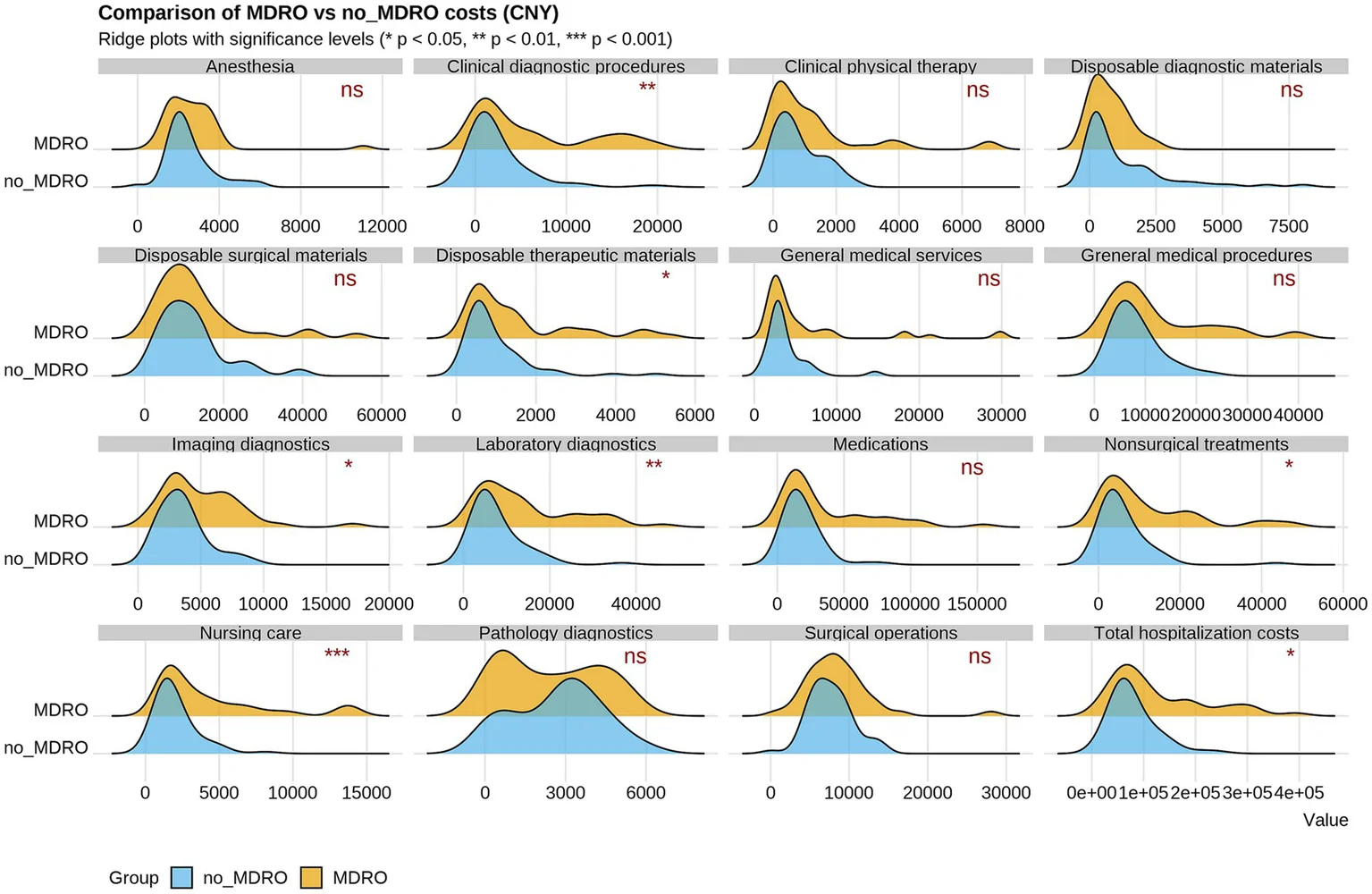
Ridge plot illustrating differences in economic burden between MDRO and non-MDRO groups. Ridge plots display the distribution of raw itemized costs (CNY). Central peaks indicate density maxima, and the width of each density curve reflects data spread. The MDRO group exhibited significantly higher costs in: Total hospitalization costs, Laboratory diagnostics, Imaging diagnostics, Clinical diagnostic procedures, Nursing care, Disposable therapeutic materials, and Nonsurgical treatments (all *p* < 0*.05,* Wilcoxon rank-sum test). No significant differences were observed in general medical services/procedures, pathology, surgery/anesthesia-related fees, medications, and diagnostic/surgical materials (*p* > 0*.05*).

Data on hospitalization expenses indicated that the total hospitalization cost in the MDRO group was 85,912.86 CNY, whereas that in the no-MDRO group was 64,746.75 CNY, with a statistically significant difference (*p < 0.05*). Among the itemized expenses, the medians of the laboratory diagnostic fees, imaging diagnostic fees, clinical diagnostic procedure fees, nursing care fees, disposable therapeutic material fees, and nonsurgical treatment fees in the MDRO group were all higher than those in the no-MDRO group, and the differences between the two groups were statistically significant (*p* < 0.05) (Figure 3, Table 3). There were no statistically significant differences between the two groups in terms of general medical service fees, general medical procedure fees, pathology diagnostic fees, surgical operation fees, anesthesia fees, medication fees, clinical physical therapy fees, disposable diagnostic material fees, or disposable surgical material fees (*p > 0.05*) (Figure 3, Table 3).

**Table 3:** Hospitalization cost comparison between the MDRO and non-MDRO groups (CNY).

Variable	Overall (*n* = 100)	Non-MDRO group (*n* = 59)	MDRO group (*n* = 41)	*u* value	*p* value
Total hospitalization costs	73,082.51 (53,334.29, 117,188.87)	64,746.75 (50,115.22, 101,542.81)	85,912.86 (59,996.13, 178,440.05)	866	0.016
General medical services	3,145.00 (2,431.00, 4,777.50)	2,978.00 (2,436.00, 4,380.00)	3,218.00 (2,278.00, 5,570.00)	1,083	0.38
General medical procedures	7,349.50 (4,997.00, 10,723.00)	6,842.00 (4,805.00, 10,294.50)	7,791.00 (5,633.00, 16,812.00)	983	0.11
Nursing care	1,753.75 (1,338.50, 3,144.00)	1,583.00 (1,219.00, 2,451.00)	2,783.00 (1,550.00, 5,951.00)	704	<0.001
Pathology diagnostics	2,894.00 (866.00, 3,906.00)	2,936.00 (1,268.00, 3,866.00)	2,326.00 (674.00, 3,982.00)	1,297	0.54
Laboratory diagnostics	6,494.75 (4,336.00, 13,304.50)	5,381.50 (3,884.50, 9,325.00)	10,318.50 (5,548.00, 22,453.50)	796	0.004
Imaging diagnostics	3,470.00 (2,501.25, 5,437.00)	3,297.50 (2,097.50, 4,152.50)	4,225.00 (2,740.00, 7,032.50)	878	0.020
Clinical diagnostic procedures	1,351.00 (587.50, 5,028.00)	1,114.00 (447.00, 2,891.50)	2,353.00 (832.00, 12,686.00)	826	0.007
Nonsurgical treatments	4,527.00 (2,558.65, 9,969.00)	3,836.00 (2,546.40, 8,210.50)	6,624.00 (2,670.00, 18,740.80)	913	0.038
Clinical physical therapy	556.00 (143.00, 1,360.00)	550.00 (102.00, 1,334.00)	644.00 (238.00, 1,360.00)	1,149	0.67
Surgical operations	7,791.75 (5,731.50, 9,656.05)	7,396.00 (5,590.00, 9,230.00)	7,879.00 (6,017.50, 10,320.00)	1,087	0.39
Anesthesia	2,307.50 (1,783.50, 3,145.00)	2,241.00 (1,799.00, 2,964.00)	2,455.00 (1,666.00, 3,280.00)	1,147	0.66
Medications	16,054.32 (10,383.53, 27,433.49)	15,998.21 (9,937.71, 23,237.11)	20,732.49 (10,873.33, 55,177.36)	940	0.059
Disposable diagnostic materials	474.08 (174.94, 1,404.78)	450.77 (174.94, 2,015.38)	555.88 (174.94, 1,053.50)	1,309	0.49
Disposable therapeutic materials	748.90 (495.27, 1,431.28)	670.02 (472.37, 1,216.68)	1,023.17 (560.19, 1,657.47)	925	0.047
Disposable surgical materials	10,011.46 (5,740.02, 14,616.48)	9,995.96 (5,772.04, 14,461.96)	10,066.96 (5,687.61, 15,133.50)	1,191	0.90

### Percentage cumulative contribution plot analysis of hospitalization cost differences

**Figure 4:**
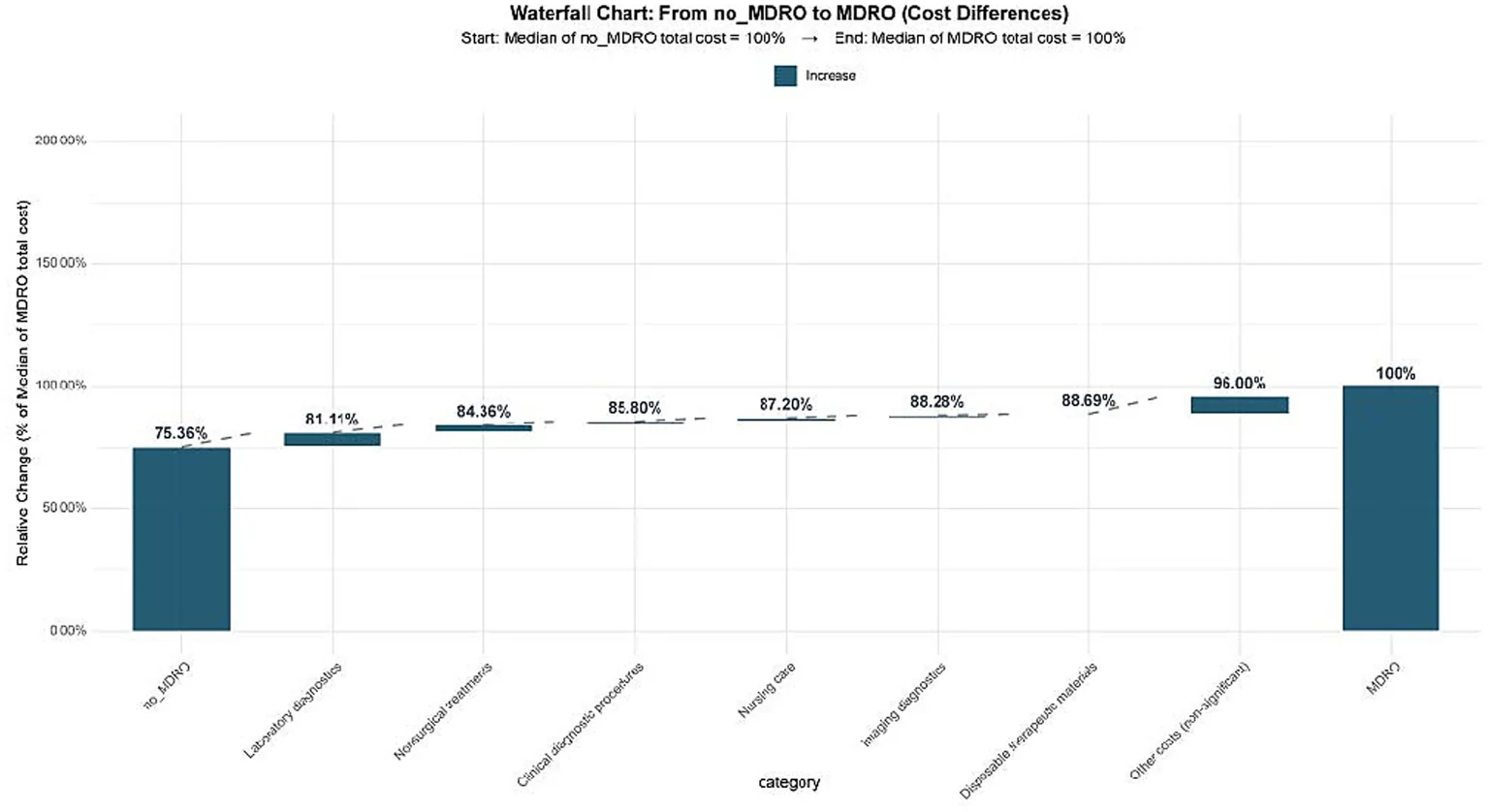
Percentage cumulative contribution plot of hospitalization cost differences between MDRO and no-MDRO groups. Using the percentage of the median total hospitalization cost of the MDRO group as the baseline (100%), a percentage cumulative contribution plot was constructed by sequentially adding the percentage contributions of the medians of each cost item with statistically significant differences between the two groups. Started from the no-MDRO group (75.36%), the contributions to the cumulative percentage of the total hospitalization cost of the MDRO group, in descending order, were as follows: Laboratory diagnostics, Nonsurgical treatments, Clinical diagnostic procedures, Nursing care, Imaging diagnostics and Disposable therapeutic materials (from 75.36 to 88.69%). The aggregate contribution of the remaining cost categories without significant differences between the two groups increased the cumulative percentage from 88.69 to 96.00%.

Using the percentage of the median total hospitalization cost of the MDRO as the baseline (100%), a percentage cumulative contribution plot (Figure 4) was constructed by sequentially adding the percentage contributions of the medians of each cost item with statistically significant differences between the two groups. The results showed that, started from the no-MDRO group (75.36%), the contributions to the cumulative percentage of the total hospitalization cost of the MDRO group, in descending order, were as follows: Laboratory diagnostics, Nonsurgical treatments, Clinical diagnostic procedures, Nursing care, Imaging diagnostics and Disposable therapeutic materials (from 75.36 to 88.69%). The aggregate contribution of the remaining cost categories without significant differences between the two groups increased the cumulative percentage from 88.69 to 96.00%.

## Discussion

In recent years, MDRO infections, as a major threat to global public health, have significantly exacerbated the burden on healthcare systems owing to their associated clinical complications and long-term consumption of medical resources. This study conducted an economic burden analysis targeting patients undergoing gastrointestinal surgery, a high-risk population for MDRO infection. The results revealed that the demographic characteristics or common comorbidities were generally similar between the MDRO and non-MDRO groups, although some clinically relevant differences were noted. However, as an observational study, we cannot rule out residual confounding. Unmeasured or inadequately adjusted confounders (e.g., severity of illness, nutritional status, preoperative antibiotic history) may still influence the cost estimates. We therefore refrain from attributing the observed cost differences solely to MDRO infection. Although some of results differ from those proposed in some previous studies that “comorbidities may increase the risk of MDRO infections (18)”, it is speculated that this may be related to the relatively limited sample size of this study or the control level of comorbidities in the included cases. However, the balance of baseline characteristics were associated with the observed differences in economic burden stem from iatrogenic factors such as antimicrobial escalation, implementation of isolation measures, and prolonged hospitalization caused by MDRO infections.

The results of this study revealed no significant differences in surgical characteristics between the two groups, but the negative impact of MDRO infection on postoperative resource utilization and clinical outcomes was particularly prominent. The prolonged ICU length of stay in patients with MDRO infection was consistent with the findings of previous studies (2, 19), which may be related to the need for more intensive life support due to severe complications such as sepsis and organ failure caused by MDROs (20). The increase in the total number of days of antimicrobial use not only reflects the restriction of MDRO infection on the choice of antimicrobial therapy but also indicates the increased difficulty of eradicating MDROs (21). A prolonged hospital stay is closely related to the need for longer isolation treatment and complication management in patients with MDRO infection (22). The hospital stays and cost of surgical patients with HAIs increase significantly (23), and the superposition of both methods further increases the burden on patients. The increased mortality and decreased cure rate highlight the essential clinical harm of MDRO infection, which echoes the conclusion that the mortality rate of MDRO infection is 2–3 times higher than that of susceptible strains (24). In addition, treatment failure or repeated illnesses may further increase medical costs, such as additional expenses for secondary surgery, advanced life support, or interdisciplinary consultation.

Notably, this study revealed that surgical characteristics did not act as a driving factor for the differences in the prognosis of MDRO infections. This suggests that, against the backdrop of homogenized surgical techniques, clinical interventions need to shift from a “surgery-centered” approach to a “patient- and infection control-centered” approach. The biological characteristics of the infection itself, the host immune status, and the implementation of infection prevention and control measures may play a dominant role in determining outcomes (25), which provides a theoretical basis for formulating perioperative management strategies in the future. In addition, the strong correlation between resource consumption indicators and clinical outcomes indicates that shortening the length of ICU length of stay may be an important breakthrough to improve prognosis, requiring ICU teams to identify high-risk patients with MDRO infections early and implement precise prevention and control measures (26).

Given the relatively small sample size in this study, conventional parametric methods for analysis could be biased or imprecise. To address this limitation, we performed a PSA using a non-parametric bootstrap method, which employs 5,000 replications to robustly estimate the sampling distributions of incremental costs and incremental effects. Total hospitalization costs were used as the cost outcome, and ICU length of stay served as the effectiveness outcome. By conducting a health economics analysis from the hospital’s perspective, we further quantified the differences between the MDRO and no-MDRO groups in terms of incremental cost-effectiveness. The bootstrap-based confidence intervals and the cost-effectiveness acceptability curve (CEAC) allowed us to characterize uncertainty without relying on normal distribution assumptions, making our findings more reliable despite the modest sample size.

Our bootstrap-based probabilistic sensitivity analysis revealed that the MDRO group was associated with significantly higher total hospitalization costs (median ΔC = 46,973 CNY, 95% CI: 18,405–78,031CNY) and a modestly longer ICU length of stay (median ΔE = 5.59 days, 95%, CI: 3.09–8.51 days). The median incremental cost-effectiveness ratio (ICER) was estimated at 8,376 CNY per additional ICU length of stay with a 95% CI ranging from 5,081 to 10,864 CNY per additional ICU length of stay.

These findings must be interpreted in the context of the clinical and economic burden of MDRO infections. Previous studies have consistently shown that MDRO infections lead to prolonged hospital stays, increased use of broad-spectrum antibiotics, and higher mortality, all of which drive up costs. Our results align with these observations: the extra ICU length of stay likely reflect the need for intensified monitoring, isolation precautions, and delayed clinical response due to limited treatment options.

The CEAC indicates that decision-makers’ willingness to pay (WTP) plays a critical role in determining whether MDRO management strategies are considered cost-effective. At the WTP threshold equal to the median ICER (8,376 CNY per additional ICU length of stay; 95% CI: 5,081–10,864 CNY per additional ICU length of stay), the probability of being attributed to MDRO is approximately 39.9% (probability of lower to upper: 6.7–90.2%). When the WTP threshold increases to 12,000 CNY per additional ICU length of stay, this probability rises to about 99.6%.

The results indicated that from a hospital perspective with a moderately high budget per additional ICU length of stay, accepting MDRO-associated extra costs may be defensible. Nevertheless, it is important to note that ICU length of stay is an intermediate process indicator rather than a final patient-centered outcome such as quality-adjusted life years (QALYs). Future research should incorporate long-term outcomes (e.g., post-discharge survival, functional status, and health-related quality of life) to provide a more complete picture of the value of interventions targeting MDRO infections.

Our analysis provides valuable evidence for hospital administrators and policymakers. The median ICER of approximately 8,376 CNY per additional ICU length of stay may serve as a benchmark for future cost-effectiveness studies on MDRO infection control. Future research should adopt a prospective design, collect comprehensive resource use data, and include patient-reported outcomes to enable full economic evaluation from a hospital perspective. Additionally, sensitivity analyses using alternative effectiveness measures (e.g., infection-related mortality, successful decolonization) would help test the robustness of our conclusions.

In conclusion, MDRO infection is associated with higher costs and prolonged ICU length of stay, with an ICER of 8,376 CNY per additional ICU length of stay. Whether this represents good value for money depends on the willingness-to-pay threshold of local healthcare decision-makers. Given the rising threat of antimicrobial resistance, further economic evaluations with longer time horizons and patient-centered outcomes are urgently needed.

Based on the significant difference in total hospitalization costs between the two groups, further analysis of the cost breakdown was conducted. Consistent with existing literature (23), surgical patients with HAIs incur significantly higher total costs, nursing service fees, laboratory fees, and consumable costs. In the present study, percentage cumulative contribution plot analysis revealed that laboratory diagnostics, non-surgical treatments, clinical diagnostic procedures, nursing care, imaging diagnostics, and disposable therapeutic materials were the main contributors to the increased total hospitalization costs in the MDRO group. The no-MDRO group baseline and the aggregate of cost categories without significant differences between the two groups together contributed only to an increase in cumulative percentage from 88.69 to 96.00%. This finding suggests that the incremental economic burden of MDRO infections is predominantly driven by direct medical resource utilization related to diagnosis, treatment, and nursing care.

Laboratory diagnostics ranked as the largest contributor, which is associated with the increased frequency of microbiological testing in MDRO infections (27), as additional pathogen identification and antimicrobial susceptibility testing may further elevate laboratory costs. Multiple studies have confirmed that patients with MDRO infections often require more intensive laboratory monitoring due to the complexity of pathogens and prolonged treatment duration, thereby significantly increasing diagnostic-related expenses (17, 28). The secondary contribution of non-surgical treatment costs reflects the extended treatment course and the need for more expensive or combination therapies in managing MDRO infections (29). The contributions of clinical diagnostic procedures and imaging diagnostics are likely attributable to recurrent imaging assessments and interventional procedures required for complications such as intra-abdominal abscesses, fistulas, or postoperative infection spread in MDRO-infected patients (30). The increases in nursing care costs and disposable therapeutic material expenses stem from longer hospital stays and greater disease complexity, which necessitate higher nursing intensity and increased use of consumable supplies (17).

Regarding cost structure characteristics, no differences were observed between the two groups in surgery-related costs such as pathology diagnostics and anesthesia, indicating that the surgical procedure itself is not a cost driver. However, MDRO infections significantly prolonged hospital stays, leading to elevated nursing care and clinical diagnostic procedure costs, consistent with reports that MDRO infections increase intensive care demands and flexible costs associated with longer hospitalization days (8). Notably, although there were no significant differences between the two groups in several routine cost categories, their aggregate contribution still raised the cumulative percentage by approximately 7.31%, suggesting a multidimensional cumulative effect of additional resource consumption caused by MDRO infections, which aligns with findings from multiple studies (31).

## Conclusion

The findings of this study demonstrate that HAIs caused by multidrug-resistant MDROs in patients undergoing gastrointestinal surgery exacerbate medical resource consumption and significantly increase hospitalization costs, prolonging hospital stay duration, ICU length of stay, and duration of antibiotic therapy. Consequently, these infections impose a significant economic burden, with laboratory diagnostics, non-surgical treatments and clinical diagnostic procedures identified as the major cost drivers. These findings underscore the urgent need to strengthen perioperative infection prevention and control measures, as well as to optimize antimicrobial stewardship strategies in clinical practice. Such interventions are essential to mitigate the financial burden on patients and to improve overall treatment outcomes.

### Limitations of the study

Several limitations of this study warrant consideration. First, this was a single-center retrospective cohort study, which may limit the generalizability of our findings and introduce selection bias. Second, we used ICU length of stay as the effectiveness measure in the cost-effectiveness analysis; as an intermediate process indicator, it does not capture differences in mortality, readmission, or long-term morbidity. Third, as an observational study without adjustment for major confounders using multivariable regression or propensity score matching, we cannot rule out residual confounding. Unmeasured or inadequately adjusted factors—such as severity of illness, nutritional status, and preoperative antibiotic history—may still have influenced the cost estimates. Fourth, utility weights for discharge outcomes (cured, improved, dead) were not incorporated; using QALYs would allow direct comparison with other healthcare interventions. Fifth, the bootstrap confidence intervals assume that the original sample is representative, and the non-parametric ellipse may be less stable with small sample sizes. Sixth, only direct in-hospital costs were included; indirect costs (e.g., productivity loss, out-of-pocket expenses) and post-discharge costs were not captured, meaning our findings reflect a hospital perspective and should not be extrapolated to societal costs. Despite these limitations, our analysis provides region-specific preliminary evidence that may inform future multicenter prospective studies and economic evaluations of MDRO infection control strategies grounded in value-based healthcare.

## Data Availability

The raw data supporting the conclusions of this article will be made available by the authors, without undue reservation.
